# Ultrasound-Assisted Extraction of Total Flavonoids from *Pteris cretica* L.: Process Optimization, HPLC Analysis, and Evaluation of Antioxidant Activity

**DOI:** 10.3390/antiox8100425

**Published:** 2019-09-24

**Authors:** Mengyang Hou, Wenzhong Hu, Aosheng Wang, Zhilong Xiu, Yusheng Shi, Kexin Hao, Xingsheng Sun, Duo Cao, Ruishan Lu, Jiao Sun

**Affiliations:** 1 School of Bioengineering, Dalian University of Technology, Dalian 116024, China; mengyanghou@yahoo.com (M.H.); zhlxiu@dlut.edu.cn (Z.X.); 2 Key Laboratory of Biotechnology and Bioresources Utilization, Ministry of Education, Dalian 116600, China; jnwangaosheng@sina.com (A.W.); haokex@sina.com (K.H.); sunxingsheng106@sina.com (X.S.); lrskarma@163.com (R.L.); sunjiao820@sina.com (J.S.); 3 College of Life Science, Dalian Minzu University, Dalian 116600, China; 4 College of Life Sciences, Yanan University, Yanan 716000, China; caoduo2013@163.com

**Keywords:** *Pteris cretica* L., flavonoids, ultrasonic-assisted extraction, optimization, response surface methodology, HPLC analysis, antioxidant activity

## Abstract

In the present work, the ultrasonic-assisted extraction (UAE) of total flavonoids (TF) from *Pteris cretica* L. was optimized by response surface methodology (RSM) on the basis of a single-factor experiment. The optimized UAE parameters were as follows: Ethanol concentration 56.74%, extraction time 45.94 min, extraction temperature 74.27 °C, and liquid/solid ratio 33.69 mL/g. Under the optimized conditions, the total flavonoids yield (TFY) was 4.71 ± 0.04%, which was higher than that obtained by heat reflux extraction (HRE). The extracts were further analyzed by HPLC, and five major flavonoids, including rutin, quercitrin, luteolin, apigenin, and luteolin-7-*O*-glucoside, were identified and quantified. Furthermore, the results of the antioxidant test showed that the TF extract obtained under optimized UAE conditions exhibited good 2,2-diphenyl-1-picrylhydrazyl radical (DPPH•) and 2,2-azino-bis(3-ethylbenzothiazoline-6-sulfonic acid) radical (ABTS^+^•), nitric oxide radical (NO•) scavenging activities, and ferrous ion (Fe^2+^) chelating capacity, with IC_50_ values of 74.49, 82.92, 89.12, and 713.41 µg/mL, respectively. Results indicated that the UAE technique developed in this work was an efficient, rapid, and simple approach for the extraction of flavonoids with antioxidant activity from *P. cretica*.

## 1. Introduction

*Pteris cretica* L., a perennial evergreen herb, belongs to the genus *Pteris* (Pteridaceae). The genus *Pteris*, geographically distributed over the tropical and subtropical regions of the world, consists of approximately 250 species, many of which have been cultivated for ornamental, culinary, and medicinal purposes [1,2]. Historically, several species of the genus *Pteris* have been used as folk medicine to treat burn injuries, indigestion, diarrhea, furuncles, eczema, apoplexy, jaundice, snakebites, and hemorrhages in China [3,4]. Due to the outstanding medicinal potential, modern investigations on the *Pteris* species have been performed extensively, which reveal that these plants contain various bioactive components, including flavonoids [5,6,7], sesquiterpenoids [3,8,9], and diterpenoids [9,10,11]. Flavonoids isolated from plants of this genus, including kaempferol, quercetin, rutin, apigenin, luteolin, and luteolin 7-*O*-sophoroside, especially contribute to various pharmacological effects, such as antibacterial [12], antioxidant [13], and anti-benign prostatic hyperplasia [14] potentials.

As is known to all, extraction is a preliminary step in phytochemical research, which is also a necessary step in pharmaceutical research. Conventional extraction methods, including maceration, decoction, heat reflux, and Soxhlet extraction, are time-consuming, costly, and inefficient [15]. Fortunately, ultrasonic-assisted extraction (UAE) makes up for those shortcomings because the cavitation, vibration, crushing, and mixing effects in media produced by ultrasound can break the cell wall and increase the mass transfer process effectively [16]. In addition, UAE can not only avoid the heat-induced destruction of bioactive ingredients, but also helps to improve the safety of products [17]. Hence, UAE as an alternative technique has been widely used for the extraction of natural bioactive compounds, especially flavonoids, for instance, flavonoids from *Medicago sativa* Linn [18], *Ampelopsis grossedentata* leaves [19], and *Olea europaea* leaves [20]. To the best of our knowledge, research on the extraction of flavonoids from *P. cretica* have not been reported.

Admittedly, extraction yield and pharmacological action depend greatly on the comprehensive effect of several various factors, including the extraction method, solvent type, extraction time, extraction temperature, pH, and liquid/solid ratio [21]. Therefore, to maximize the extraction yield of bioactive substances and pharmacological effects, optimization of the extraction process is quite essential. Recently, response surface methodology (RSM) as an advanced chemometric tool has been frequently applied to the optimization of the extraction process [22]. RSM design can reduce the number of experimental trials, resulting in lower reagent consumption and less laboratory work. Additionally, RSM design yields a mathematical model to account for the reciprocal influence of various independent variables [23]. Box–Behnken design (BBD), one type of RSM, is easier to interpret and perform in comparison with other designs [24].

The present study is an attempt to establish an efficient UAE technique for total flavonoids from *P. cretica*. First, the effects of four independent variables, including ethanol concentration, extraction time, extraction temperature, and liquid/solid ratio, on the yield of total flavonoids were investigated. Second, the reciprocal actions of independent variables were investigated followed by the optimization of the UAE process using RSM. Third, a heat reflux extraction experiment was carried out to verify the efficiency of the UAE method developed. Fourth, a comparison of flavonoid profiles of the extracts obtained by UAE under optimized conditions and heat reflux extraction (HRE) was performed by HPLC. In addition, the antioxidant activity of extracts obtained by optimized UAE was evaluated by 2,2-diphenyl-1-picrylhydrazyl radical (DPPH•), 2,2-azino-bis(3-ethylbenzothiazoline-6-sulfonic acid) radical (ABTS^+^•), nitric oxide radical (NO•) scavenging activities, and Fe^2+^ chelating activity.

## 2. Materials and Methods

### 2.1. Chemicals and Reagents

Rutin, quercetin, luteolin, apigenin, and luteolin-7-*O*-glucoside standards were obtained from Shanghai Yuanye Bio-Technology Co., Ltd. (Shanghai, China). HPLC grade trifluoroacetic acid (TFA) and methanol were obtained from Aladdin Reagent Co., Ltd. (Shanghai, China). Ascrobic acid, 2,2-diphenyl-1-picrylhydrazyl (DPPH) and 2,2-azino-bis(3-ethylbenzothiazoline-6-sulfonic acid) (ABTS), ethylenediaminetetraacetic acid disodium salt (EDTA-2Na), sodium nitroprusside (SNP), potassium persulfate (K_2_S_2_O_8_), and trolox were obtained from Sigma-Aldrich (St. Louis, MO, USA). Other chemicals used (analytical grade) were bought from Kemio Chemical Reagent Co., Ltd. (Tianjin, China).

### 2.2. Plant Material

*P. cretica* was collected from Kunming City, Yunnan Province, China. A voucher specimen (FWJ-20180301) was deposited in the College of Life Science, Dalian Minzu University, Dalian, China. The plant material was dried under natural ventilation until reaching constant weight. The plant material was then powdered and stored in an air-tight container for further use.

### 2.3. Optimization of UAE of TF from P. cretica

#### 2.3.1. Single-Factor Experiments

The UAE of TF from *P. cretica* was performed in a water-bath sonicator (KQ5200DE, 40 kHz frequency and 200 W nominal power, Kunshan Ultrasonic Instrument Co., Jiangsu, China). To study the influence of ethanol concentration on the total flavonoids yield (TFY), 5.0 g of pretreated samples were placed into glass conical flasks (250 mL) and soaked with ethanol solvents (varying from 30–80%, *v/v*), the time for extraction was set at 30 min, the temperature applied for extraction was set at 70 °C, and the liquid/solid ratio was set at 30 mL/g. To select the optimum extraction time, different extraction times (10, 20, 30, 40, 50, and 60 min) were tested under the conditions of ethanol concentration 60%, extraction temperature 70 °C, and liquid/solid ratio 30 mL/g. To choose the best extraction temperature, different extraction temperatures (40, 50, 60, 70, 80, and 90 °C) were studied under the following conditions: Ethanol concentration 60%, extraction time 40 min, and liquid/solid ratio 30 mL/g. Finally, the effect of liquid/solid ratios of 15, 20, 25, 30, 35, and 40 mL/g on the extraction yield was evaluated under the following conditions: Ethanol concentration 60%, extraction time 40 min, and extraction temperature 70 °C. After each UAE, the extraction solution was filtered, and the sample residue was re-extracted twice under the same extraction conditions. The combined extractive solution was then concentrated in vacuo and the extract was stored at −20 °C for further analysis.

#### 2.3.2. RSM Design

Based on the results of single-factor experiments, the UAE of TF from *P. cretica* was further optimized by RSM. In this work, the BBD with four variables (ethanol concentration *X*_1_, extraction time *X*_2_, extraction temperature *X*_3_, and liquid/solid ratio *X*_4_) at three levels (−1, 0, 1) was carried out to evaluate the effect between every two variables on the response value (TFY *Y*). The BBD procedure of RSM resulted in a total of 29 randomized experiments, and a quadratic model used to analyze experimental data was shown as follows:

(1) $$Y=\alpha_{0}+\sum_{i=1}^{4}\alpha_{i}X_{i}+\sum_{i=1}^{4}\alpha_{\mathrm{ii}}X_{i}{}^{2}+\sum_{i=1}^{3}\sum_{j=i+1}^{4}\alpha_{\mathrm{ij}}X_{i}X_{j}$$

where *Y* is the response value; *α*_0_ refers to the intercept; *α*_i_, *α*_ii_, and *α*_ij_ refer to the linear, quadratic, and interactive coefficients, respectively; *X*_i_ and *X*_j_ represent the independent variables (i ≠ j).

### 2.4. Conventional Heat Reflux Extraction (HRE)

A comparative study between the HRE and UAE was carried out to estimate the efficiency of the UAE process established in this work. The HRE of TF from *P. cretica* was performed under the optimized UAE conditions with slight modifications. In brief, 5.0 g of the pre-prepared sample was extracted three times under reflux with 170 mL of 57% ethanol at 75 °C, each time for 2 h. After the HRE, the extract was processed as the method described in Section 2.3.1.

### 2.5. Measurement of Total Flavonoids Content (TFC)

The total flavonoids content (TFC) was measured using the aluminium chloride colourimetric method reported by with slight modifications [25]. First, to 0.5 mL of the sample solution, 0.15 mL of NaNO_2_ (5%, *w/v*) was added. After the mixture was stirred for 5 min, 0.15 mL of (10%, *w/v*) AlCl_3_ was added. After another 5 min, 1 mL of NaOH (1 M) was added. The solution was then made up to a final volume of 5 mL by the addition of ultrapure water. After complete addition, the reaction mixture was further incubated at ambient temperature for 20 min, and the absorbance was immediately recorded at 510 nm with an UV-vis Spectrophotometer (UV-2600, Shimadzu, Kyoto, Japan). Rutin was used as a reference, and the TFC was calculated according to the regression equation: y=11.2736x−0.0028 (*R*^2^ = 0.9997, final rutin concentration 18.12–72.48 µg/mL), where *y* is the absorbance, *x* is the content (µg/mL), and the TFY was calculated by the equation given below:

(2) $$\mathrm{Extraction}\;\mathrm{yield}\;(\%)=\frac{C\times V}{W}\times 100$$

where *C* represents the TFC (mg/mL); *V* represents the total volume of extractive solution (mL); and *W* represents the dry weight of plant material (mg).

### 2.6. HPLC Analysis

An HPLC system (Shimadzu Co., Kyoto, Japan) equipped with a multi-channel pump (LC-20AD) and a diode array detector (SPD-M20A) was used to analyze the extracts from *P. cretica* obtained by UAE under optimized conditions and HRE. A YMC-Pack ODS-A column (5 μm, 250 mm × 4.6 mm id; YMC Co., Tokyo, Japan) was employed for all separations at 25 °C. The mobile phase was composed of 0.5% TFA in ultrapure water (solvent A) and methanol (solvent B). The elution procedure was as follows: 0–25 min, 25–45% B; 25–40 min, 45–65% B; 40–60 min, 65–90% B; 60–65 min, 90–25% B, with a flow rate of 0.5 mL/min. Detection was carried out at the wavelength of 254 nm. The flavonoids in extracts from *P. cretica* were identified on the basis of comparison of the retention time and ultraviolet spectrum of standards, and the quantification of flavonoids was performed by the external standard method.

### 2.7. Method Validation

The method was validated for linearity, limit of detection (LOD), limit of quantification (LOQ), precision (inter-day and intra-day precision), stability, and accuracy following the International Conference on Harmonization (ICH) guideline and the previous reports [26,27].

Linearity was examined through the triplicate analysis of mixed standard solutions at six different concentrations, and the calibration curves were constructed by linear regression analysis of the integrated peak areas (*y*) versus concentrations (*x*). LOD and LOQ for each analyte under the HPLC conditions were determined at the signal-to-noise ratio (S/N) of 3 and 10, respectively.

Intra-day and inter-day variations were chosen to evaluate the precision of the developed HPLC method. Intra-day precision was assessed by six injections of a mixed standard solution within a single day, and inter-day precision was validated with the mixed standard solution used above once a day for 3 consecutive days. The precision of this method was expressed as relative standard deviation (RSD). Stability was evaluated by analyzing the same sample at 0, 2, 4, 6, 8, and 12 h at room temperature. The accuracy test was performed by the standard addition method. The authentic standards at three different concentration levels (low, medium, and large) were added to the sample, then extracted and quantified based on the established procedures. The recovery was counted according to the following formula:

(3) $$\mathrm{Recovery}\;(\%)=\frac{\mathrm{total}\;\mathrm{detected}\;\mathrm{amount}-\;\mathrm{original}\;\mathrm{amount}}{\mathrm{added}\;\mathrm{amount}}\times 100$$

### 2.8. Evaluation of Antioxidant Activity

#### 2.8.1. Assay of DPPH• Radical Scavenging Activity

The DPPH• radical scavenging capacity of total flavonoid extracts of *P. cretica* was investigated on the basis of the method reported by Shukla et al. [28] with a minor modification. Briefly, 0.8 mL of the DPPH solution (0.1 mM in ethanol) was mixed with 2.4 mL of total flavonoid extract solution at different concentrations (25–250 µg/mL in ethanol). The reaction mixture was then shaken well and incubated at room temperature for 30 min. The absorbance of the reaction mixture was recorded at 517 nm. Ascrobic acid was used as a positive control in this work. The percentage of DPPH• radical scavenging was calculated using the following equation:

(4) $$\mathrm{Radical}\;\mathrm{scavenging}\;\mathrm{activity}\;(\%)=\frac{A_{0}-A_{1}}{A_{1}}\times 100$$

where *A*_0_ represents the absorbance of the reaction system without the extract and the *A*_1_ represents the absorbance of the reaction system in the presence of the extract. The test was carried out in triplicate, and the IC_50_ was defined as the concentration of the extract that resulted in a 50% inhibition of DPPH• radical.

#### 2.8.2. Assay of ABTS^+^• Radical Scavenging Activity

The method reported by Awe et al. [29] was adopted for the assay of ABTS^+^• radical scavenging activity. First, ABTS was dissolved in phosphate buffered saline (PBS, 0.01 M, pH 7.4) to a 7 mM concentration. The ABTS solution was then mixed with an equal volume of K_2_S_2_O_8_ solution (2.45 mM) in the dark at room temperature to produce ABTS^+^•. After 16 h, the ABTS^+^• solution was adjusted to a suitable absorbance (0.70 ± 0.02) using water at 734 nm. To 2.0 mL of the prepared ABTS^+^• solution, 0.5 mL of various concentrations of extract solution (25–150 µg/mL) was added. The reaction mixture was then incubated in the dark at room temperature. A total of 10 mins later, the absorbance of the mixture was immediately recorded at 734 nm. Trolox was used as a positive control in this experiment, and the percentage of ABTS^+^• radical scavenging was calculated using the Equation (4).

#### 2.8.3. Assay of NO• Radical Scavenging Activity

The assay of NO• radical scavenging activity was carried out following the procedure of Shukla et al. [28] with a minor modification. Briefly, 5 mM of SNP in PBS solution (0.01 M, pH 7.4) was prepared and mixed immediately with 2.0 mL of various concentrations of *P. cretica* extract (25–250 µg/mL). After 150 min of incubation at room temperature, 1.0 mL of Griess reagent (1% sulfanilamide in 2% H_3_PO_4_ and 0.1% naphthylethylenediamine dihydrochloride) was added. The absorbance of the chromophore formed during the reaction was then recorded at 546 nm. Ascrobic acid was used as a positive control in this work, and the percentage of NO• radical scavenging was calculated using Equation (4).

#### 2.8.4. Assay of Fe^2+^ Chelating Activity

The ferrous ion chelating activity of the extract was measured using the modified method reported by Tohma et al. [30]. In brief, this assay was carried out by mixing 1.0 mL of extract solution (50–1600 µg/mL) with 0.1 mL of FeCl_2_ solution (2.0 mM), 0.2 mL of ferrozine solution (5.0 mM) and 2.7 mL of deionized water. After 10 min of incubation at room temperature, the absorbance of reaction mixture was recorded at 562 nm, and EDTA-2Na was used as a positive control. The Fe^2+^ chelating activity was calculated according to Equation (4).

### 2.9. Statistical Analysis

All the experiments were performed in triplicate, and the results were shown as mean ± standard deviation (SD). A variance analysis (ANOVA) is used and the comparison of the averages is carried out by the test of Duncan, and differences were regarded as significant when the *p* < 0.05. The Design-Expert version 10 software (Stat-Ease Inc., Minneapolis, MN, USA) was employed for the RSM design and statistics analysis.

## 3. Results

### 3.1. Single-Factor Experiments

#### 3.1.1. Effect of Ethanol Concentration on the TFY

**Figure 1:**
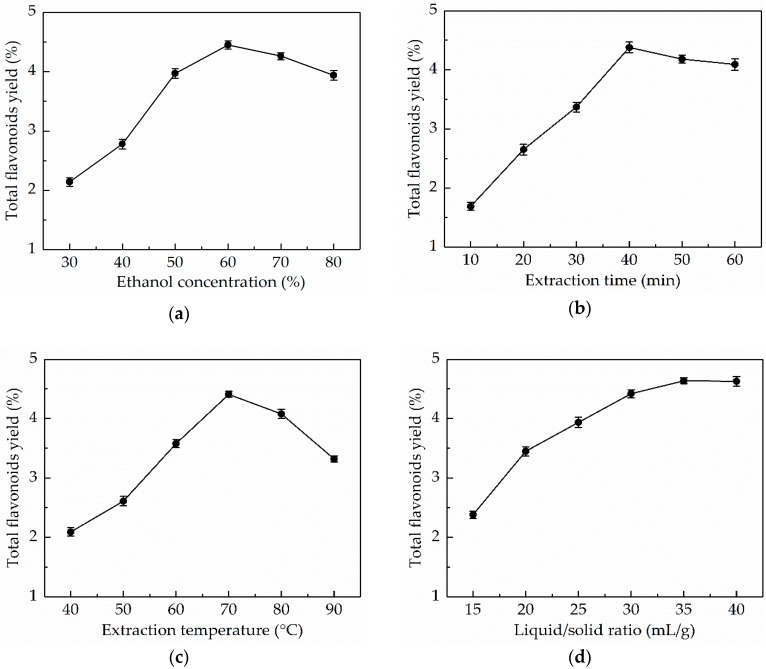
Effects of four independent variables on the total flavonoids yield (TFY): (**a**) Ethanol concentration; (**b**) extraction time; (**c**) extraction temperature; and (**d**) liquid/solid ratio.

In general, aqueous ethanol is an excellent solvent for the extraction of flavonoids from plants, and the selection of an appropriate ethanol concentration is a crucial step to improve extraction yield. In the present work, the impact of ethanol concentration on the TFY was studied first. As shown in Figure 1a, the TFY increased dramatically when the ethanol concentration varied from 30% to 60%. However, the extraction yield exhibited a downward trend when the ethanol concentration was above 60%, which was possibly due to the fact that higher ethanol concentration could more easily lead to dissolution of alcohol-soluble impurities [31]. Therefore, 60% ethanol was chosen as the best extraction solvent.

#### 3.1.2. Effect of Extraction Time on the TFY

In this work, the influence of extraction time (10–60 min) on the TFY was researched. As shown in Figure 1b, the initial increase of extraction time (10–40 min) resulted in an obvious improvement in the TFY. The extraction yield decreased slightly with the further increase in extraction time, which could be ascribed to the fact that longer extraction time will lead to an increase in the amount of dissolved impurities and the degradation of some flavonoids [32]. Thus, 40 min was regarded as the optimum extraction time.

#### 3.1.3. Effect of Extraction Temperature on the TFY

There is no doubt that temperature is a significant factor influencing the extraction yield. Hence, in order to obtain the optimum extraction temperature, UAE processes were performed at 40, 50, 60, 70, 80, and 90 °C, respectively, and the results were shown in Figure 1c. There was a significant increase in the TFY (varying from 2.09 ± 0.07% to 4.41. ± 0.05%) with increasing extraction temperature from 40 to 60 °C. However, at higher extraction temperature (more than 60 °C), the TFY declined. One of the possible reasons for this was that the structure of the flavonoids was destroyed under high temperature [33]. Thus, 60 °C was selected as the optimal extraction temperature.

#### 3.1.4. Effect of Liquid/Solid Ratio on the TFY

The effect of liquid/solid ratio (15–40 mL/g) on the TFY was in Figure 1d. The TFY increased with the increasing liquid/solid ratio and reached a maximum (4.64 ± 0.05%) at 35 mL/g. Whereas, extraction yield reached a plateau phase when the liquid/solid ratio continued to rise above 35 mL/g. A high liquid/solid ratio would give rise to an increase in the cost of recovery. Hence, the optimal liquid/solid ratio was set at 35 mL/g.

### 3.2. Optimization of UAE Process by RSM

#### 3.2.1. Model Fitting and Statistical Analysis

Due to the association between variables, BBD with four factors and three levels was employed to optimize the individual parameters. BBD matrix and response values were listed in Table 1. Subsequently, statistical regression analysis of experimental data was carried out, and a second-order polynomial equation yielded was represented as below:

(4) $$\begin{array}{cc} Y= & 4.59-0.068X_{1}+0.27X_{2}+0.27X_{3}+0.015X_{4}\\ & -0.23X_{1}X_{2}-0.29X_{1}X_{3}+0.098X_{1}X_{4}+0.14X_{2}X_{3}\\ & -0.19X_{2}X_{4}-0.098X_{3}X_{4}-0.54X_{1}{}^{2}-0.38X_{2}{}^{2}\\ & -0.55X_{3}{}^{2}-0.33X_{4}{}^{2} \end{array}$$

where *X*_1_, *X*_2_, *X*_3_, and *X*_4_ are the ethanol concentration, extraction time, extraction temperature, and liquid/solid ratio, respectively. *Y* is the predicted value of TFY.

#### 3.2.2. Response Surface Analysis

The three-dimensional (3D) response surface images illustrate the mutual influence of any two independent variables on the dependent variable while keeping other variables at the 0 level, and the shape of the 3D response surface plots provides information on the influence degree. To be specific, elliptical or saddle shapes of the 3D surfaces usually represent the interaction between two independent variables on the dependent variable, which is relatively significant, whereas a gently sloping surface indicates that the interaction between two factors on the dependent variable is relatively mild [34,35].

**Figure 2:**
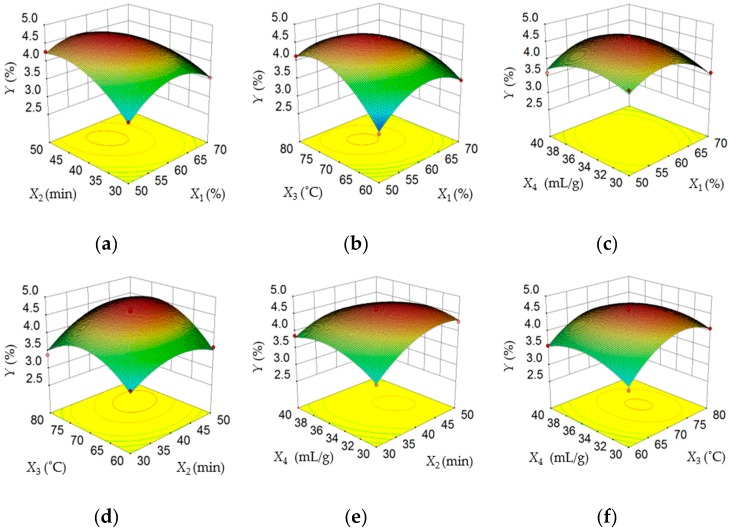
Response surface plots showing interaction between different variables (*X*_1_: Ethanol concentration; *X*_2_: Extraction time; *X*_3_: Extraction temperature; and *X*_4_: Liquid/solid ratio) on the TFY.

Figure 2a–f offered a visual interpretation of the interactions between two variables (*X*_1_*X*_2_, *X*_1_*X*_3_, *X*_1_*X*_4_, *X*_2_*X*_3_, *X*_2_*X*_4_, and *X*_3_*X*_4_) on the response variable (*Y*). Figure 2a–c showed the influences of ethanol concentration (*X*_1_) with extraction time (*X*_2_) and extraction temperature (*X*_3_) on the extraction yields of TF, respectively. The initial increase of *X*_1_ (50% to about 57%) led to an increase in TFY and followed by a decline thereafter (about 57% to 70%). Similarly, as could be seen from Figure 2a,d,e, a rapid rise in TFY was obtained when *X*_2_ varied from 30 to about 46 min, then the extraction yield of TF was decreased slowly with increasing extraction time. Figure 2b,d,f indicated that with an increase of *X*_3_ from 60 to about 74 °C, the TFY increased quickly, followed by a slight decline with further increase in *X*_3_. According to Figure 2c,e,f, it could be found that a maximum level of TFY was obtained at a *X*_4_ of 34 mL/g. The graphs also indicated that the influence of *X*_4_ on the TFY is insignificant (*p* > 0.05). In addition, as evident from Figure 2c, the interaction effect of *X*_1_ and *X*_4_ had a insignificant influence on the TFY from *P. cretica* (*p* > 0.05).

#### 3.2.3. Validation of the Optimized Model

The optimal conditions for UAE of TF from *P. cretica* obtained by RSM were as follows: Ethanol concentration (*X*_1_) 56.74%, extraction time (*X*_2_) 45.94 min, extraction temperature (*X*_3_) 74.27 °C, and liquid/solid ratio (*X*_4_) 33.69 mL/g. Under optimized UAE conditions, the theoretical TFY was 4.74%. Subsequently, in order to prove the reliability of the prediction model, the confirmatory experiments were carried out under the optimized extraction conditions. Confirmatory experiments gave a satisfactory result that the TFY was 4.71 ± 0.04%, which was well-matched with theoretical value. Therefore, it could be concluded that the regression model was appropriate for the optimization of the UAE process of TF from *P. cretica*.

In this study, HRE was also performed to confirm the superiority of UAE of TF from *P. cretica*. The results demonstrated that the TFY obtained by UAE under optimized conditions was higher than that obtained by HRE (3.48 ± 0.05%). Additionally, a lot of previous literatures have reported that UAE gave a higher TFY obtained by UAE when compared with HRE. This phenomenon was believed to be caused by cavitation, vibration, crushing, mixing, and other comprehensive effects produced by ultrasound, which enhanced the extractability of flavonoids [36].

### 3.3. Method Validation for Quantitative Analysis of Five Flavonoids

Table 3 listed the linear equation, correlation coefficient (*R*^2^), linear range, LOD, and LOQ of each compound determined. It could be found that all calibration curves showed good linearity (*R*^2^ ≥ 0.9995) within the tested concentration range. For these compounds, the LOD values ranged from 0.014 to 0.094 µg/mL, while the LOQ values ranged from 0.16 to 1.31 µg/mL. The results of precision, stability, and accuracy tests were shown in Table 4. All RSD values of the intra-day and inter-day precision ranged from 0.94–2.43% and 1.87–2.51%, respectively, which indicated good precision of the developed method. The results of the stability test showed that the RSD values of peak areas of the five compounds were ≤2.56%, which indicated that the sample was stable for 12 h at room temperature. According to the calculation, the mean recoveries of five compounds ranged from 98.93 to 101.3%, with RSD values ranging from 0.85 to 2.19%, which demonstrated a good accuracy of the developed method. In sum, the verification tests demonstrated that the developed method was feasible for the simultaneous quantification of five compounds from *P. cretica*.

### 3.4. Analysis of Flavonoids in the Extracts by HPLC

**Figure 3:**
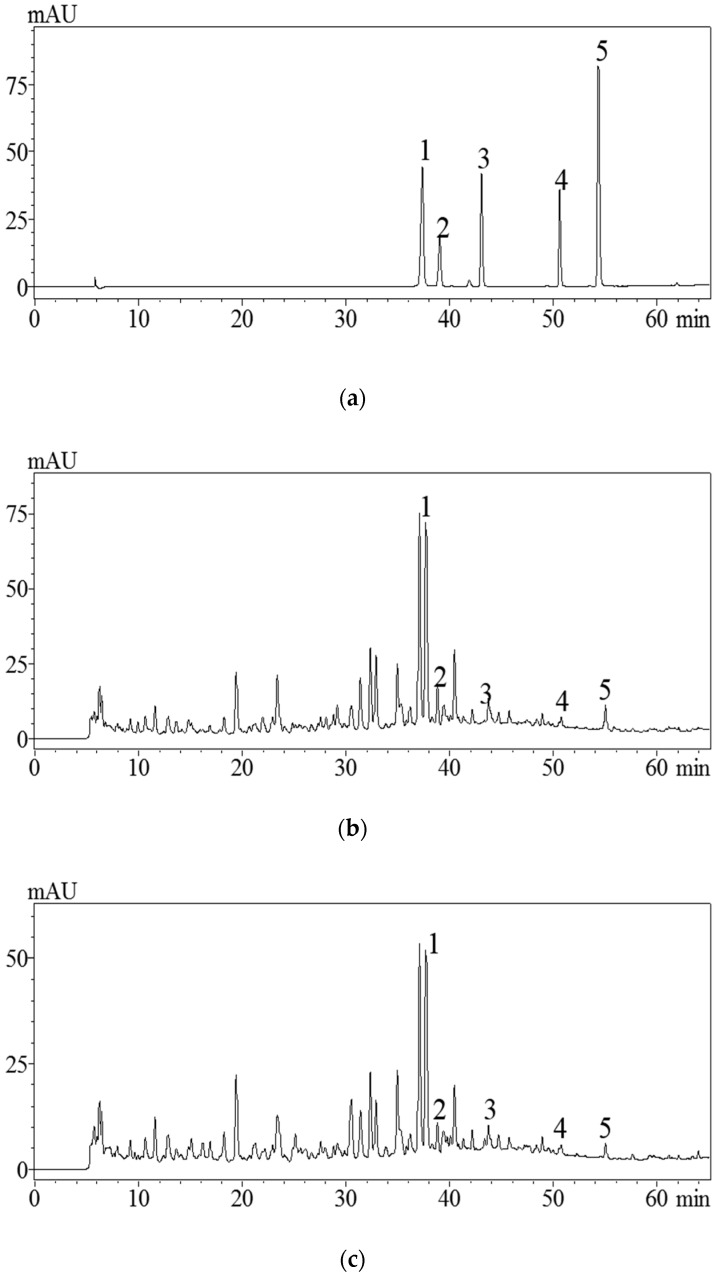
HPLC profiles of standards mixture (**a**) and *P. cretica* extract obtained by optimized ultrasonic-assisted extraction (UAE) (**b**). *P. cretica* extract obtained by heat reflux extraction (HRE) (**c**). Peaks (1): Luteolin-7-*O*-glucoside, (2) rutin, (3) quercitrin, (4) luteolin, and (5) apigenin.

Without any doubt, pharmacological activity of *P. cretica* depends on its chemical constituents. Therefore, flavonoid profiles in the *P. cretica* extracts were analyzed by HPLC. Figure 3 indicated that the HPLC chromatograms of extracts obtained by UAE and HRE showed highly similar characteristics. A total of five flavonoids, including luteolin-7-*O*-glucoside, rutin, quercetin, luteolin, and apigenin, were identified, and their elution times were 37.340, 39.021, 43.031, 50.568, and 54.306 min, respectively. Based on the results of HPLC quantitative analysis, the contents of the five flavonoids (mg/g plant material, dry weight) were in the following descending order: Luteolin-7-*O*-glucoside (3.237 ± 0.015 mg/g) > rutin (1.483 ± 0.009 mg/g) > apigenin (0.803 ± 0.013 mg/g) > luteolin (0.679 ± 0.007 mg/g) > quercitrin (0.582 ± 0.011 mg/g). However, the contents of five flavonoids extracted by HRE were all lower compared to the UAE: Luteolin-7-*O*-glucoside (2.862 ± 0.005 mg/g) > rutin (1.133 ± 0.007 mg/g) > apigenin (0.659 ± 0.011 mg/g) > luteolin (0.486 ± 0.009 mg/g) > quercitrin (0.574 ± 0.013 mg/g). Thus, it could be concluded that the UAE was a promising extraction method for the separation of bioactive flavonoids from *P. cretica* in the future. To our knowledge, this work was the first quantitative analysis of these five flavonoids in *P. cretica*.

### 3.5. Antioxidant Activity

#### 3.5.1. DPPH• Radical Scavenging Activity

**Figure 4:**
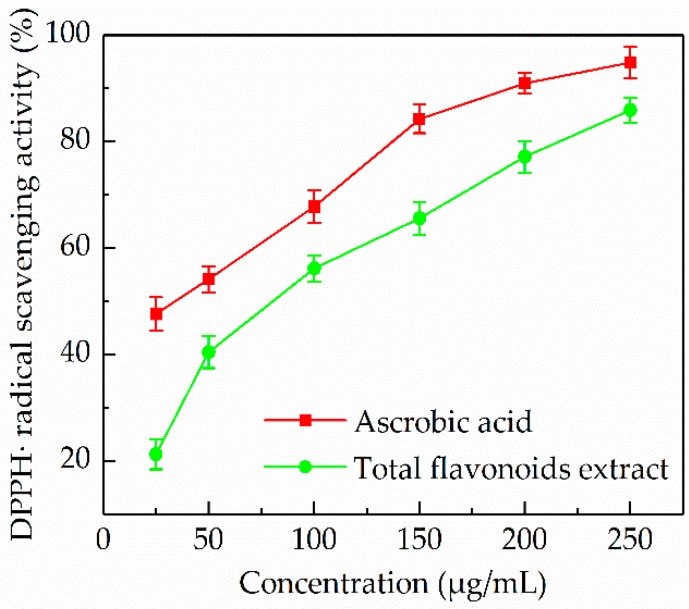
Scavenging activity of *P. cretica* extract obtained by optimized ultrasound-assisted extraction against DPPH• radical compared with ascorbic acid.

The DPPH• radical scavenging activity assay is a fairly common method to evaluate the antioxidant activity of natural products [37]. A comparison of the ascorbic acid and the *P. cretica* extract obtained by optimized UAE was performed, and the results are shown in Figure 4. It was observed that the DPPH• radical scavenging effect of the *P. cretica* extract was increased with increasing concentration. The *P. cretica* extract at the test concentrations of 25, 50, 100, 150, 200, and 250 µg/mL showed 21.23 ± 2.78, 40.45 ± 3.04, 56.17 ± 2.45, 65.56 ± 3.14, 77.13 ± 2.98, and 85.94 ± 2.37% DPPH• radical scavenging, respectively. The IC_50_ value of the *P. cretica* extract was 74.49 µg/mL, while for ascorbic acid it was 35.19 µg/mL. The results indicated that the UAE extract of *P. cretica* had a good potential for scavenging DPPH• radical. It has been reported that DPPH• radical scavenging activity of plant extracts is ascribable to the presence of flavonoids [38].

#### 3.5.2. ABTS^+^• Radical Scavenging Activity

**Figure 5:**
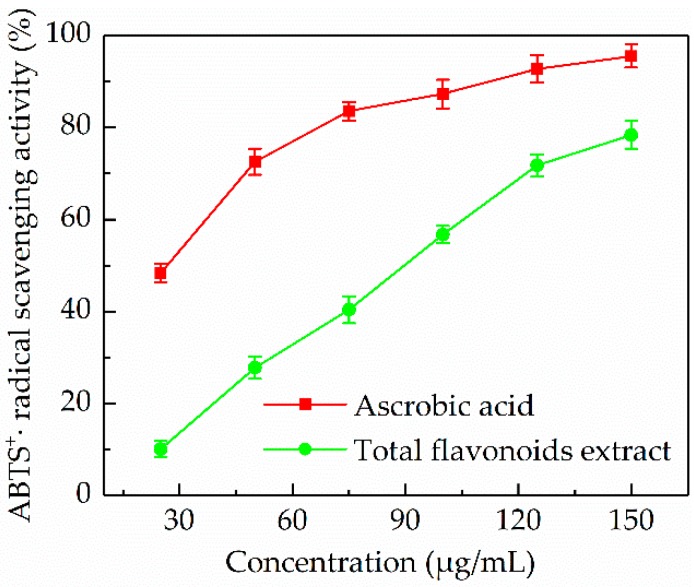
Scavenging activity of the *P. cretica* extract obtained by optimized ultrasound-assisted extraction against ABTS^+^• radical compared with trolox.

One of the most widely used organic radicals for the determination of antioxidant activity of natural products is the radical cation derived ABTS [39]. The results of ABTS^+^• radical scavenging activity of the *P. cretica* extract at different concentrations (25–150 µg/mL) were shown in Figure 5. It was found that the *P. cretica* extract had a scavenging activity on the ABTS^+^• radical in a dose dependent manner. The IC_50_ value of standard trolox was 30.10 µg/mL, while the IC_50_ value of the *P. cretica* extract was 82.92 µg/mL. A 150 µg/mL of the *P. cretica* extract exhibited 78.41 ± 2.97% inhibition. From these results, it could be stated that the *P. cretica* extract was a good ABTS^+^• radical scavenger.

#### 3.5.3. NO• Radical Scavenging Activity

**Figure 6:**
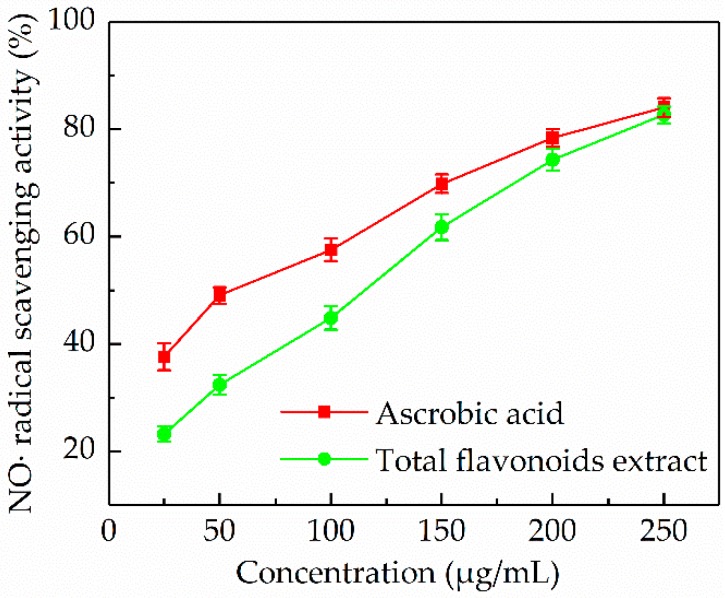
NO• radical scavenging activity of the *P. cretica* extract obtained by optimized ultrasound-assisted compared with ascorbic acid.

Despite the potential health benefits of NO• radical, its role in causing oxidative damage is becoming progressively prominent [40]. In this work, the scavenging effect of the *P. cretica* extract on NO• radical was studied. Figure 6 showed that the *P. cretica* extract within the concentration range tested (25–250 µg/mL) exhibited NO• radical scavenging activity in a dose-dependent manner. Especially, the *P. cretica* extract exhibited a maximum percentage scavenging of 82.71 ± 1.54%, which was comparable to the NO• radical scavenging activity of ascorbic acid. The IC_50_ value of ascorbic acid was 51.60 µg/mL, and for the *P. cretica* extract it was 89.12 µg/mL. Results indicated that *P. cretica* might serve as an excellent NO• radical scavenger due to the high level of flavonoids. It has been reported that the flavonoids are very powerful NO• radical scavengers [41].

#### 3.5.4. Fe^2+^ Chelating Activity

**Figure 7:**
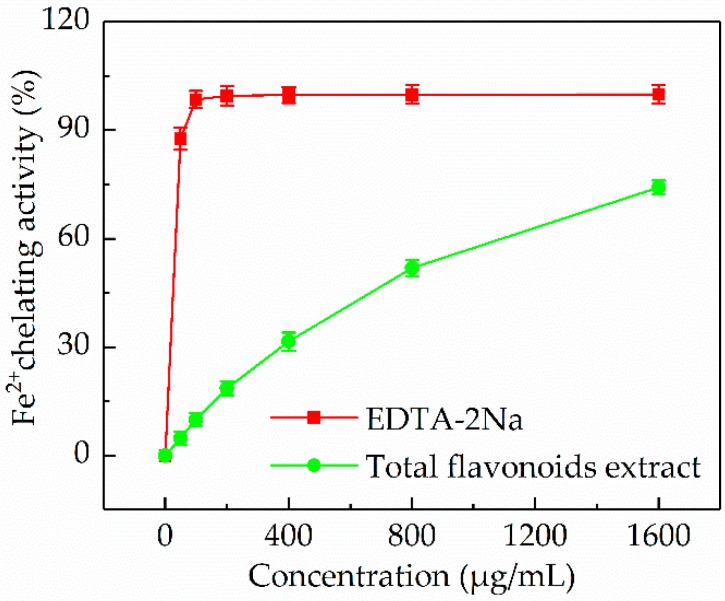
Fe^2+^ chelating activity of the *P. cretica* extract obtained by optimized ultrasound-assisted compared with EDTA-2Na.

It has been reported that Fe^2+^ is a powerful pro-oxidant among various metal ions and Fe^2+^ chelation may prevent oxidative damage through retarding metal-catalyzed oxidation and inhibiting ROS production [42]. In this work, the Fe^2+^ chelating activity of the *P. cretica* extract was determined and compared with a chelating standard (EDTA-2Na). As shown in Figure 7, at different test doses of 50, 100, 200, 400, 800, and 1600 µg/mL, the Fe^2+^ chelating activity of the *P. cretica* extract was 4.74 ± 1.77, 9.98 ± 1.89, 18.65 ± 1.86, 31.56 ± 2.48, 51.87 ± 2.17, and 74.21 ± 1.83%, respectively, with an IC_50_ value of 713.41 µg/mL. According to these results, it could be concluded that the *P. cretica* extract had certain Fe^2+^ chelating activity.

## 4. Conclusions

In this work, the UAE was proved to be a sample, rapid, and effective procedure for the extraction of TF from *P. cretica*, and the process parameters optimized by RSM were as follows: Ethanol concentration 56.74%, extraction time 45.94 min, extraction temperature 74.27 °C, and liquid/solid ratio 33.69 mL/g. Under optimized UAE conditions, the TFY was 4.71 ± 0.04%, which was higher than that obtained by HRE. Moreover, HPLC analysis indicated that luteolin-7-*O*-glucoside, rutin, quercitrin, luteolin, and apigenin were the predominant flavonoids in *P. cretica*. It was also found that UAE is more suitable than HRE for the extraction of these compounds. In addition, the *P. cretica* extract obtained through the optimized UAE method exhibited good DPPH•, ABTS^+^•, and NO• scavenging activities. The results of the present wok should contribute to the further and deeper investigation of *P. cretica*, which might be used as a novel source of natural antioxidants.

## Figures and Tables

**Table 1:** Box–Behnken design matrix and experimental values for the TFY.

Run	*X*_1_ (%)	*X*_2_ (min)	*X*_3_ (°C)	*X*_4_ (mL/g)	*Y* (%)
1	60 (0)	40 (0)	70 (0)	35 (0)	4.64
2	70 (1)	30 (−1)	70 (0)	35 (0)	3.56
3	60 (0)	30 (−1)	70 (0)	40 (1)	3.87
4	60 (0)	30 (−1)	80 (1)	35 (0)	3.39
5	60 (0)	40 (0)	80 (1)	30 (−1)	4.08
6	50 (−1)	40 (0)	70 (0)	40 (1)	3.60
7	50 (−1)	40 (0)	60 (−1)	35 (0)	2.95
8	60 (0)	40 (0)	70 (0)	35 (0)	4.61
9	50 (−1)	50 (1)	70 (0)	35 (0)	4.27
10	50 (−1)	40 (0)	70 (0)	30 (−1)	3.97
11	70 (1)	40 (0)	70 (0)	40 (1)	3.63
12	60 (0)	40 (0)	80 (1)	40 (1)	4.04
13	60 (0)	40 (0)	60 (−1)	40 (1)	3.58
14	60 (0)	40 (0)	70 (0)	35 (0)	4.61
15	60 (0)	50 (1)	80 (1)	35 (0)	4.23
16	60 (0)	40 (0)	70 (0)	35 (0)	4.62
17	60 (0)	40 (0)	60 (−1)	30 (−1)	3.23
18	60 (0)	50 (1)	60 (−1)	35 (0)	3.61
19	60 (0)	30 (−1)	70 (0)	30 (−1)	3.38
20	70 (1)	40 (0)	60 (−1)	35 (0)	3.46
21	60 (0)	50 (1)	70 (0)	40 (1)	4.01
22	70 (1)	40 (0)	80 (1)	35 (0)	3.48
23	60 (0)	40 (0)	70 (0)	35 (0)	4.48
24	70 (1)	50 (1)	70 (0)	35 (0)	3.64
25	50 (−1)	40 (0)	80 (1)	35 (0)	4.13
26	60 (0)	50 (1)	70 (0)	30 (−1)	4.28
27	70 (1)	40 (0)	70 (0)	30 (−1)	3.61
28	50 (−1)	30 (−1)	70 (0)	35 (0)	3.27
29	60 (0)	30 (−1)	60 (−1)	35 (0)	3.33

The fitness and adequacy of the regression model were evaluated using a variance analysis (ANOVA), and results were given in Table 2. *F*-value (41.92) and *p*-value (<0.0001) of the regression model indicated that the established model was very significant. Whereas, *F*-value (3.28) and *p*-value (0.1317) of the lack of fit indicated that the lack of fit was not significant as compared with the pure error. In addition, the determination coefficient (*R*^2^) obtained for this model was 0.9767, implying that the model could satisfactorily fit the variability of the TFY. The predicted *R*^2^ of 0.8764 was in reasonable agreement with the adjusted *R*^2^ of 0.9534, implying that the predicted values were highly consistent with the experimental values. In this study, the linear coefficients (*X*_1_, *X*_2_, and *X*_3_), quadratic term coefficients (*X*_12_, *X*_22_, *X*_32_, and *X*_42_), and the cross product coefficients (*X*_1_*X*_2_, *X*_1_*X*_3_, *X*_2_*X*_3_, and *X*_2_*X*_4_) had statistically significant effects on the TFY (*p* < 0.05).

**Table 2:** The analysis of variance for the second-order polynomial model.

Source	Sum of Squares	Df	Mean Square	*F*-Value	*p*-Value
Model	6.28	14	0.45	41.92	<0.0001
*X*_1_	0.055	1	0.055	5.11	0.0403
*X*_2_	0.87	1	0.87	81.73	<0.0001
*X*_3_	0.85	1	0.85	79.22	<0.0001
*X*_4_	0.0027	1	0.0027	0.25	0.6233
*X*_1_ *X*_2_	0.21	1	0.21	19.77	0.0006
*X*_1_ *X*_3_	0.34	1	0.34	31.43	<0.0001
*X*_1_ *X*_4_	0.038	1	0.038	3.55	0.0804
*X*_2_ *X*_3_	0.078	1	0.078	7.32	0.0170
*X*_2_ *X*_4_	0.14	1	0.14	13.49	0.0025
*X*_3_ *X*_4_	0.038	1	0.038	3.55	0.0804
*X*_12_	1.90	1	1.90	177.64	<0.0001
*X*_22_	0.95	1	0.95	88.74	<0.0001
*X*_32_	1.95	1	1.95	182.59	<0.0001
*X*_42_	0.70	1	0.70	65.06	<0.0001
Residual	0.15	14	0.011		
Lack of fit	0.13	10	0.013	3.28	0.1317
Pure error	0.016	4	0.004		
Cor total	6.43	28			
*R* ^2^	0.9767				
Adjusted *R*^2^	0.9534				

**Table 3:** Linear regression, limit of detection (LOD), and limit of quantification (LOQ) of the five tested compounds.

Analytes	Linear Equation	*R* ^2^	Linear Range (µg/mL)	LOD (µg/mL)	LOQ (µg/mL)
luteolin-7-*O*-glucoside	y=73104x−11077	0.9995	5.00–100	0.051	0.67
Rutin	y=62441x+21414	0.9998	5.00–100	0.094	1.31
Quercitrin	y=135884x+32168	0.9997	2.50–100	0.042	0.44
Luteolin	y=146356x−48920	0.9998	1.00–100	0.014	0.16
Apigenin	y=86719x−22486	0.9999	1.00–100	0.026	0.34

**Table 4:** Precision, stability, and recovery of the five tested compounds.

Analytes	Precision (RSD, %)		Stability (RSD, %)	Recovery	
	Intra-Day	Inter-Day		Mean Recovery (%)	RSD (%)
Luteolin-7-*O*-glucoside	1.34	2.51	2.56	99.67	0.85
Rutin	2.11	1.87	2.48	100.4	1.48
Quercitrin	0.94	2.02	1.89	101.3	2.19
Luteolin	1.52	1.98	2.05	98.93	1.83
Apigenin	2.43	2.16	2.33	99.21	2.07
